# From binding to networks: methods for identifying transcription factor targets in plant systems

**DOI:** 10.3389/fpls.2026.1760251

**Published:** 2026-04-13

**Authors:** Adam T. Sumner, Bastiaan O. R. Bargmann, David C. Haak

**Affiliations:** School of Plant and Environmental Sciences, Virginia Polytechnic Institute and State University, Blacksburg, VA, United States

**Keywords:** functional genomics, gene regulatory networks, plant genomics, target identification, transcription factors

## Abstract

Transcription factors (TFs) orchestrate gene expression programs by binding regulatory DNA sequences and modulating transcription of target genes. Identifying TF–target gene relationships is fundamental to understanding plant development, stress responses, and metabolic regulation. However, determining which genes a TF regulates remains technically challenging. This review provides a decision-oriented framework, that integrates experimental and computational plant TF–target identification. Placing emphasis on plant-specific constraints and practical method selection to guide researchers from initial TF discovery through comprehensive network characterization. We compare biochemical approaches (EMSA, Y1H), genome-wide mapping methods (ChIP-seq, DAP-seq, CUT&Tag), expression profiling techniques (RNA-seq on mutants and overexpression lines), and computational prediction tools (GENIE3, PTFSpot, ConnecTF). Critical trade-offs are discussed, between binding potential and functional regulation, throughput and resolution, and between different model and non-model plant systems. Finally, we highlight emerging technologies including high-throughput enhancer screening, single-cell approaches, and machine learning-based prediction platforms that promise to accelerate functional characterization of plant TFs and their regulatory networks.

## 1 Introduction

The study of plant transcription factors (TFs) has profound implications for both basic plant biology and agricultural applications. From a fundamental perspective, TFs provide insights into how plants coordinate complex developmental programs, respond to environmental challenges, and maintain cellular homeostasis. Understanding TF function reveals the molecular mechanisms underlying this coordination. From an applied perspective, TF manipulation represents a powerful tool for crop improvement. For example, overexpression of DREB1A/CBF TFs has enhanced drought and freezing tolerance in Arabidopsis and multiple crops, GRF4 variants have increased rice grain yield, and NAC family TFs have been used to improve stress tolerance and grain nutritional quality in cereals (Behnam et al., 2007; Hu et al., 2006; Kasuga et al., 1999; Li et al., 2016, 2022). Because individual TFs often regulate large sets of genes controlling complex traits such as plant biomass, stress tolerance, disease resistance, or nutritional quality, they offer opportunities to engineer multiple beneficial characteristics simultaneously (Haak et al., 2017). Plant TFs have the potential to be leveraged for agricultural trait improvements and understanding how TFs interact with target genes to coordinate plant growth and development is a crucial step.

Identifying the specific targets that a TF binds to and regulates is essential for understanding how plants control gene expression to coordinate biological processes. TFs orchestrate transcription by recognizing and binding to the regulatory elements in the target genes’ proximal promoter and distal regulatory regions, resulting in activation or repression of the target genes by recruiting transcriptional cofactors to the site. The discovery of these TF–target interactions uncovers the function of the TF and the cellular processes that it affects.

Identifying a TF’s targets is only one aspect of its characterization. TFs typically function within complex gene regulatory networks (GRNs), which are systems of interacting TFs, target genes, and cofactors that collectively control gene expression programs in a cell. Analyzing TFs through the lens of GRNs reveals their conditional, context−dependent regulation of target genes and clarifies how regulatory interactions change across tissues, developmental stages, and environments. In these networks, multiple TFs and regulatory elements act together to form regulatory cascades and feedback loops that shape the dynamics and robustness of gene expression. Placing a TF within its respective network provides insight into how its activity is modulated by other network components and how it participates in co−regulatory relationships within specific biological pathways. Uncovering GRNs is an ambitious goal of plant TF studies, and method selection is crucial for properly elucidating new networks and building on previously identified ones.

**Figure 1:**
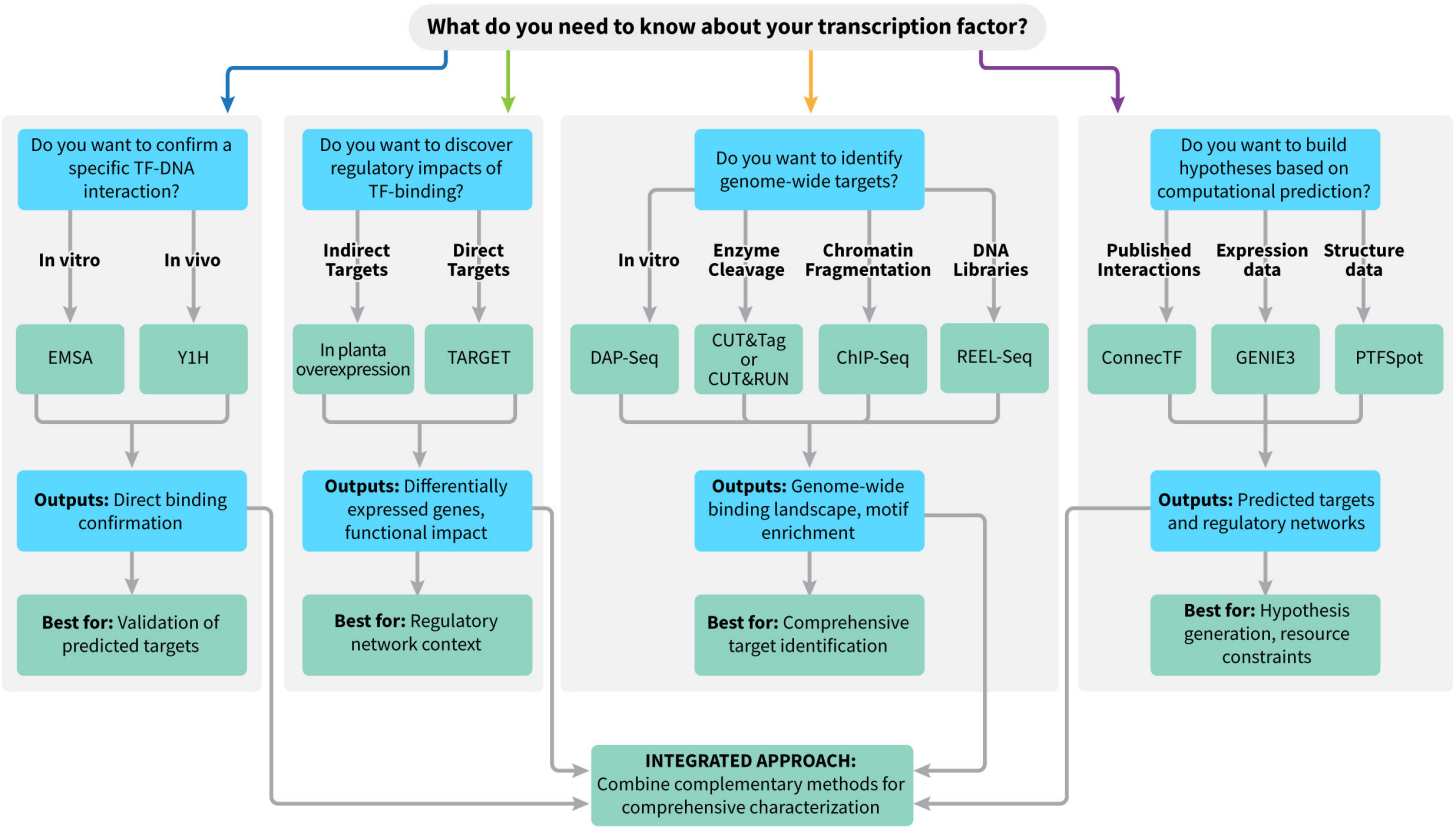
Integrated Workflow for Transcription Factor Target Identification in Plant Systems. The decision-tree flowchart summarizes experimental and computational strategies for characterizing transcription factor (TF) targets, highlighting which methods are optimal depending on the biological question and available resources. Each branch outlines key differences between techniques and their outputs and specific advantages. The diagram emphasizes combining complementary approaches for comprehensive TF target discovery and validation.

While recent reviews have provided comprehensive overviews of transcriptional regulatory landscapes and gene regulatory network (GRN) inference in crops emphasizing multi-omics integration and computational network reconstruction (Huo et al., 2024; Jyoti et al., 2024), our review focuses specifically on experimental and computational methods for identifying TF targets and how to combine them into a practical, decision oriented workflow tailored to plant systems. We emphasize method integration with concrete selection criteria summarized in **Figure 1** and **Table 1** to guide future studies rather than primarily surveying GRN modeling and multi-omics frameworks, highlighting plant specific constraints, tradeoffs, and integration opportunities for both model and non-model species.

**Table 1:** Comprehensive comparison of transcription factor target identification methods.

Method	Binding type	Resolution	Chromatin context	Functional data	Throughput	Primary advantage	Primary limitation	Reference	Cost	Time investment	Required equipment
EMSA	Direct	N/A	No	No	Low	Direct binding confirmation	Low throughput, *in vitro* only	Garner and Revzin (1981)	Low	Low (protein purification, probe design)	Electrophoresis system
Y1H (Gene-centered)	Direct	Motif level	No	No	Low	Direct binding confirmation	Low throughput, already know target gene	Li and Herskowitz (1993)	Low-medium	Medium (yeast work, construct cloning)	Yeast culture facilities, cloning and transformation tools
Y1H (TF-centered)	Direct	Motif level	No	No	High	Discovers binding motifs, unbiased	High false positives, labor intensive	Ji et al., 2018	Medium	High (library construction, screening)	Yeast robotics helpful, plate readers, molecular biology lab
ChIP-PCR	Direct	100–500 bp	Yes	No	Low	*In vivo* chromatin context, low cost	Limited to candidate regions	Gilmourt & Lis (1986)	Low-medium	Medium (crosslinking, IP optimization)	Standard cell/tissue culture, sonicator, qPCR machine
ChIP-Seq	Direct	100–300 bp	Yes	No	High	Comprehensive genome-wide mapping	Requires transgenic line with tagged TF	Johnson et al. (2007)	High	High (ChIP optimization, NGS library prep, analysis)	ChIP-capable lab, NGS platform or access, compute cluster
ChIP-nexus	Direct	Single bp	Yes	No	High	Single bp resolution, precise motif discovery	Requires transgenic line with tagged TF, complex analysis	He et al. (2015)	High	Very High (complex protocol and bioinformatics)	Advanced ChIP lab, NGS, high-performance computing
REEL-Seq	Direct	Single bp	No	No	Very High	Ultra-high resolution mapping of TF binding motifs	*In vitro* only, dependent on synthetic library	Zhao et al. (2020)	High	High (library design, sequencing depth)	Oligo synthesis, NGS platform, bioinformatics pipeline
DamID	Direct	1–2 kb	Yes	No	High	Does not require antibody, works *in vivo*	Lower spatial resolution, requires transgenesis	Greil et al. (2006)	Medium-high	High (transgenesis, methylation mapping)	Transgenic facility, microarrays or NGS, methylation assays
CUT&Tag	Direct	10–50 bp	Yes	No	High	Low input	Optimization needed per cell type	Kaya-Okur et al. (2019)	Medium-high	Medium-high (bead handling, nuclei prep)	Magnetic beads, NGS platform, low-input library workflow
CUT&RUN	Direct	10–50 bp	Yes	No	High	Fast, low background, low input	Optimization needed, nuclei isolation required	Skene et al. (2018)	Medium-high	Medium-high (permeabilization, nuclease control)	CUT&RUN reagents, NGS platform, standard molecular lab
DAP-Seq	Direct	50–100 bp	No	No	High	No transgenic line needed	Requires protein purification, no chromatin context	Bartlett et al. (2017)	Medium-high	High (protein expression, DNA library prep)	Protein purification system, NGS platform
RNA-Seq (Constitutive)	Indirect	Gene level	Yes	Yes	High	Global regulatory effects, identifies all targets	Cannot distinguish direct *vs* indirect	Tu et al. (2022)	Medium-high	Medium (library prep, analysis)	NGS platform, bioinformatics resources
RNA-Seq (Inducible)	Indirect	Gene level	Yes	Yes	High	Temporal control, reversible induction	Requires construct generation, may have artifacts	Curtis and Grossniklaus (2003)	Medium-high	High (construct design, induction kinetics)	NGS platform, bioinformatic analysis, induction system
TARGET	Direct (Inferred)	Gene level	Yes	Yes	High	Separates direct *vs* indirect targets	Requires protoplast system, lower throughput	Bargmann et al. (2013)	Medium-high	High (protoplasts, nuclear import system)	Protoplast isolation setup, NGS
PTFSpot	Predicted	Motif level	No	No	Very High	90% accuracy, no experimental cost	Requires TF structure, prediction only	Gupta et al. (2024)	Very low	Low (structural modeling, software)	Computing cluster, structural and prediction software
GENIE3	Inferred	Gene level	N/A	Yes	Very High	Network inference from expression alone	Correlation not causation, prediction only	Huynh-Thu and Geurts (2018)	Low	Medium (parameter tuning, validation)	Compute cluster, RNA-seq datasets, GENIE3 software or scripts
ConnecTF	Integrated	Gene level	N/A	Yes	Very High	Integrates multiple data types, validated targets	Requires extensive prior data, limited to studied TFs	Brooks et al. (2021)	Low	Medium (data integration, curation)	Web platform access, curated TF datasets, expression and binding data
ENTRAP-seq	Direct (Inferred)	Motif level	Yes	No/Optional	High	Captures TF-associated nascent transcription at binding sites	Newer method, specialized library prep and analysis	Alamos et al., 2025	Medium-high	High (nascent RNA capture, custom analysis)	Cell/tissue culture, nascent RNA workflow, NGS, compute
PIVOT	Direct	Gene level	Yes	Yes	High	Integrates perturbation and expression to refine TF targets	Requires well-controlled perturbation and high-quality expression data	Lowensohn et al. (2025)	Medium-high	High (experimental design, modeling)	Perturbation system (e.g., CRISPR or inducible lines), RNA-seq, compute
scRNA-seq	Indirect	Single cell	Yes	Yes	High	Resolves cell-type-specific regulatory programs and TF activity	High cost per cell, complex library prep and analysis	Tang et al. (2009)	Very high	Very high (single-cell workflow, complex analysis)	Single-cell platform, NGS, high-performance computing
ATAC-seq	Indirect	50–150 bp	Yes	No	High	Profiles genome-wide chromatin accessibility linked to TF activity	Does not directly identify specific TF–DNA contacts	Buenrostro et al. (2015)	Medium-high	Medium-high (nuclei prep, tagmentation)	ATAC-seq reagents (Tn5), NGS platform, standard molecular lab

Approaches are categorized by binding detection type and spatial resolution. Chromatin context refers to whether the method detects binding in native chromatin (*in vivo*) versus purified components (*in vitro*). Functional data indicates whether the method provides gene expression changes. Throughput reflects the number of genes/targets analyzed simultaneously. Primary advantages and limitations highlight key considerations for method selection.

A TF with unknown biological function but predicted DNA-binding can be identified through various methods: differential gene expression analysis, genome-wide association studies linking TF loci to phenotypic traits, yeast one-hybrid screens using promoter sequences of genes of interest, comparative genomics revealing conserved TFs, motif discovery from co-regulated gene sets, or chromatin accessibility assays that suggest TF activity patterns. These discovery approaches identify candidate TFs that require subsequent functional validation to determine their regulatory targets and mechanisms.

Understanding how a TF-of-interest functions within regulatory networks requires refining technical approaches that can both identify targets and the TF’s position within a GRN. These approaches address questions relevant to the characterization of a TF: What genes are regulated by the TF? What are the sites that a TF recognizes and binds to? What are the direct versus indirect targets of the TF? How does the TF fit into regulatory networks?

Several techniques have been developed in pursuit of answering the questions posed above. Over the past several decades, TF–target identification methods have evolved from individual binding assays toward genome-wide and computational approaches. Early work relied on biochemical assays such as electrophoretic mobility shift assays (EMSA) and yeast one-hybrid (Y1H) in the 1980s–1990s to validate individual TF–DNA interactions, followed by chromatin-based methods like Chromatin Immunoprecipitation (ChIP)-PCR in the 1990s that placed binding events into native chromatin context. The 2000s–2010s saw the emergence of genome-wide binding approaches (ChIP-seq, Affinity Purification [DAP]-seq, Cleavage Under Targets and Tagmentation [CUT&Tag]) and expression-based methods such as RNA-seq and Transient Assay Reporting Genome-wide Effects of Transcription factors (TARGET), enabling systematic identification of putative targets and functional responses. More recently, modern computational tools that leverage network-inference tools and single-cell multi-omics platforms have enabled integration of binding, expression, and chromatin data to build comprehensive gene regulatory network models in plants. Throughout this review, we distinguish between methods that quantify binding potential: where and how strongly a TF can bind DNA; and those that measure functional regulation: changes in gene expression or chromatin state resulting from TF activity. Binding alone is not sufficient to infer regulation, and functional readouts without binding information cannot resolve direct from indirect targets; full TF characterization requires integrating both. Below, we discuss the array of approaches available to study TFs, providing in-depth descriptions, explanations, applications, limitations, and integration options across approaches (**Figure 1**). We start with the long-standing individual protein-DNA binding techniques that were developed to provide fundamental insights into the direct binding of a TF to a gene’s regulatory elements. Next, this review leads into genome-wide mapping of binding sites that allow a full scope of the regions in the genome bound by a TF. We then delve into gene expression analysis approaches that give insights into the TF’s regulation patterns of the transcriptome. Finally, the section on computational techniques brings TF target characterization into the contemporary bioinformatics age where valuable information is gained even without conducting wet lab experiments. Each experimental approach offers answers to these TF characterization questions, but the full characterization of a TF is only achieved by careful selection and integration of multiple techniques.

### 1.1 Plant-specific challenges in TF–target identification

Plant genomes present several challenges for TF–target identification that are less pronounced in many non-plant systems. Large, often duplicated, and highly repetitive genomes complicate read mapping and peak calling, and regulatory elements can reside far from their target genes, making it difficult to confidently link TF binding events to specific loci. Many crops are polyploid, with closely related homeologs and paralogs that are hard to distinguish at the sequence level, further complicating assignment of TF binding and expression effects to individual gene copies.

Plant tissues are also highly heterogeneous, with diverse cell types and developmental zones within a single organ, so bulk assays can obscure cell-type-specific TF functions or dilute localized regulatory events below detection. In addition, TF binding specificity and target sets can diverge substantially across species, limiting how far motifs and regulatory models can be transferred from Arabidopsis or other model plants to crops. Finally, many agronomically important species still lack the high-quality reference genomes, annotations, and genetic tools that underpin TF–target mapping in model systems. These plant-specific constraints strongly influence which TF–target identification methods are feasible and how their results should be interpreted in both model and non-model species.

## 2 Individual protein-DNA binding

Early TF research was conducted through approaches that defined individual protein-DNA interactions, also referred to as one-to-one binding assays. While modern technologies have transformed the field of TF characterization by taking genome-wide approaches, these early approaches remain valuable for those seeking to confirm the binding of a TF to a specific region of DNA. This section examines four foundational TF-DNA-binding analyses: EMSA, selection and amplification binding assays (SAAB), Y1H assays, and ChIP-PCR.

### 2.1 Electrophoretic mobility shift assay

EMSA is based on the principle that protein-DNA complexes migrate more slowly through polyacrylamide gels than free DNA due to their increased molecular weight and altered charge-to-mass ratio (Garner and Revzin, 1981; Hellman and Fried, 2007). The technique involves incubating a purified TF with a radiolabeled or fluorescently labeled DNA probe containing the suspected binding site, followed by gel electrophoresis to separate bound and unbound DNA. EMSA offers several advantages for TF characterization. It provides evidence of direct protein-DNA binding interactions and can determine binding specificity through competition experiments with unlabeled DNA (Stowell et al., 2021). Site-directed mutagenesis of the candidate binding site, followed by EMSA, can provide a straightforward test of whether specific nucleotides are required for TF–DNA complex formation. EMSA is particularly valuable for confirming binding sites identified through other approaches that necessitate validation of the binding pairing (Kan et al., 2021; Niu et al., 2020). The traditional EMSA approach has been extended to variants including Capillary Electrophoretic Mobility Shift Assay (CEMSA), Immunodepletion EMSA (IDEMSA), and Multiplexed Competitor EMSA (MC-EMSA), which adapt the basic gel-shift principle for higher throughput, improved quantification, or specific mechanistic questions (Dyer and Herzogl, 1995; Foulds and Etzkorn, 1998; Smith and Humphries, 2009).

SAAB combines EMSA with iterative selection of bound sequences to define TF–DNA-binding specificity (Blackwell and Weintraub, 1990). In this approach, TF protein is incubated with a library of randomized oligonucleotides, protein–DNA complexes are separated from free DNA by EMSA, and the shifted band is excised and PCRamplified. Repeating this bind–separate–amplify cycle enriches high affinity binding sequences, which are then sequenced and aligned to derive a consensus binding motif and position weight matrix. The method’s power was demonstrated in the characterization of the maize INDETERMINATE1 (ID1) protein, where SAAB successfully identified the 11 bp consensus binding motif for the previously uncharacterized ID domain (IDD), establishing this plant-specific TF family and laying the foundation for understanding IDD-mediated gene regulation across plant species (Kozaki et al., 2004). SAAB’s unbiased nature makes it particularly valuable for characterizing TFs with completely unknown DNA-binding preferences, where candidate target sequences cannot be predicted from sequence homology or structural modeling. Once SAAB identifies the consensus binding motif, researchers can use site-directed mutagenesis of individual amino acids within the TF, followed by standard EMSA validation with the defined consensus sequence, to systematically map which protein regions are essential for DNA recognition and binding affinity.

Despite its utility as a routine method to confirm direct TF binding, traditional EMSA has several limitations that must be considered. The technique is inherently low-throughput, examining one protein-DNA interaction at a time, making it unsuitable for genome-wide studies or when interaction partners are still unknown. EMSA also is performed under *in vitro* conditions that lack DNA accessibility context and other regulatory factors present in living cells, potentially missing important regulatory interactions or detecting false-positive binding events. The technique also requires high-quality purified recombinant TF protein, typically produced in heterologous expression systems such as bacteria, yeast, or *in vitro* translation, which maintains proper folding and, to the extent possible, the post-translational modifications present in native plant proteins. Additionally, EMSA provides no information about the functional consequences of binding or the indirect targets of the TF.

### 2.2 Yeast one-hybrid assay

With a similar goal to EMSA, characterizing individual TF-DNA binding interactions, Y1H assays provide a powerful heterologous, *in vivo* approach for testing binding within a cellular context. Y1H involves introducing a DNA sequence of interest fused to a reporter gene along with a TF-fusion construct into yeast cells (Li and Herskowitz, 1993; Deplancke et al., 2004). If the TF binds to the DNA sequence, transcription of the reporter gene(s) (most commonly HIS3 and/or LacZ) is activated by the reporter’s promoter being simultaneously bound by an activation domain that has been fused to the TF, allowing for detection of positive interactions through growth selection on histidine-deficient media and/or colorimetric detection of β-galactosidase activity. Compared with the *in vitro* approach of EMSA, Y1H offers the advantage of taking place in a eukaryotic cellular environment (Fuxman Bass et al., 2016).

TF-Centered Y1H represents an innovative advancement that reverses the traditional Y1H screening approach to enable comprehensive motif discovery for individual TFs (Ji et al., 2018). This approach employs the TF of interest as bait while screening against a random short DNA sequence library containing all possible 7–8 base pair combinations, effectively transforming Y1H from a gene-centered validation tool into a TF-centered discovery platform. The technique constructs a comprehensive prey library by inserting random DNA sequences into the histidine reporter vector, enabling systematic identification of all potential binding motifs recognized by a specific TF without prior knowledge of target sequences. This approach has proven highly effective for plant TF characterization, as demonstrated by the identification of six distinct motifs recognized by Arabidopsis bZIP proteins, including five previously unknown binding sequences that expanded understanding of bZIP regulatory specificity (Ji et al., 2014). TF-centered Y1H represents a significant evolution of the classical Y1H system, especially for those seeking to study recognition sites of specific uncharacterized TFs.

While Y1H provides valuable insights into TF-DNA direct binding, it still has several important limitations. A significant technical challenge is reporter auto-activation in the absence of TF binding, which can lead to false positives in a substantial fraction of screens and requires careful bait and prey screening optimization. The assay has the advantage of being performed in a cellular environment, but the utilized yeast cells lack plant-specific cofactors, chromatin modifications, or protein partners required for some interactions. Just as with EMSA, indirect transcriptional regulation that occurs in the plant is also unable to be monitored. Additionally, the assay provides no information about the gene expression patterns or functional relevance of the interactions detected, leaving questions about the strength and magnitude of TF regulatory effects. Y1H is ideally utilized as a useful approach to validate one-to-one direct binding of a TF to cis-regulatory regions *in vivo*.

### 2.3 Chromatin immunoprecipitation PCR

ChIP-PCR represents an early chromatin immunoprecipitation approach that preceded well-known genome-wide ChIP-seq technology, enabling targeted analysis of TF binding to specific genomic loci within native chromatin context. The technique involves crosslinking proteins to DNA in living cells, fragmenting the chromatin, immunoprecipitating the TF of interest using specific antibodies or an epitope tag antibody, and using PCR amplification to detect TF binding at predetermined target sequences (Gilmourt and Lis, 1986). Unlike EMSA and Y1H, which examine individual protein-DNA interactions in synthetic contexts, ChIP-PCR provides the critical advantage of studying TF binding within the natural chromatin environment, accounting for chromatin accessibility, epigenetic modifications, and the presence of other regulatory proteins.

While ChIP-PCR offers the advantage of studying TF binding in native chromatin, it shares the low-throughput limitation of EMSA and Y1H by examining only predetermined target sequences rather than enabling genome-wide discovery of binding sites. ChIP-based methods also require high-quality, highly specific antibodies against the TF of interest or transgenic lines expressing epitope-tagged TFs, which involves substantial time, cost, and experimental effort. This makes the approach particularly challenging in non-model plant species. Additionally, ChIP-PCR provides no direct information about the functional consequences of TF binding or the magnitude of regulatory effects on gene expression. These limitations led to the development of ChIP−seq, which retains the chromatin-context advantages of ChIP−PCR while enabling higher-throughput, genome−wide discovery of TF binding sites; however, any ChIP technology does not resolve the functional consequences of TF binding on gene expression.

## 3 Genome-wide mapping of protein-DNA binding

The development of high-throughput sequencing technologies has revolutionized TF research by enabling genome-wide mapping of protein-DNA interactions. Unlike the approaches that examine individual binding events, deep sequencing-based methods can simultaneously identify thousands of TF binding sites across entire genomes. This section examines genome-wide binding methods organized into two categories: (i) chromatin-based *in vivo* approaches (ChIP-seq, CUT&Tag, Cleavage Under Targets and Release Using Nuclease [CUT&RUN], DNA adenine methyltransferase identification [DamID]) that map TF binding within living cells, maintaining native chromatin context, and (ii) *in vitro* approaches (DAP-seq, Restriction Enzyme-mediated Enhanced Liberation [REEL]-seq) that test purified TFs against genomic DNA libraries, enabling high-throughput screening without antibody requirements. These assays map where a TF is physically associated with DNA *in vivo* or *in vitro*, defining binding potential and candidate target loci, but they do not by themselves demonstrate that a gene is transcriptionally regulated by the TF.

### 3.1 Chromatin immunoprecipitation sequencing

ChIP-seq is a major advancement for the previously mentioned ChIP-PCR technique. It leverages high-throughput sequencing of TF-associated DNA fragments to identify binding locations down to a resolution of 150–300 base pairs (Johnson et al., 2007). Unlike one-to-one binding assays, this approach enables the ability to generate comprehensive binding profiles for TFs, revealing their suite of binding targets in a relatively unbiased manner without the requirement of preselected genes of interest. Just as with other ChIP approaches, ChIP-seq accounts for chromatin accessibility, epigenetic alterations, and the presence of other regulatory proteins that influence TF binding.

ChIP-nexus advances traditional ChIPseq by using exonuclease digestion to trim TF–DNA fragments down to precise crosslinking sites, achieving near single nucleotide resolution (He et al., 2015). A single adaptor-ligation step adds random barcodes to each molecule, allowing PCR duplicates to be collapsed and therein, reducing technical noise. This yields complementary peak pairs on opposite strands that precisely define binding boundaries and help distinguish closely spaced sites, improving motif enrichment and mapping of TF binding relative to regulatory elements. For plant TF characterization, ChIP-nexus offers advantages by eliminating background noise common in plant ChIP experiments due to chromatin complexity, providing superior motif enrichment with regulatory sequences found directly at peak centers, and enabling precise mapping of TF binding relative to transcription start sites and other regulatory elements.

ChIP-seq also has limitations that necessitate complementation with other approaches. ChIP-seq captures both direct TF–DNA binding and indirect associations mediated by protein–protein interactions with other DNA-binding factors. This characteristic represents both an advantage: enabling detection of functional TF complexes and co-regulatory interactions that occur *in vivo*, and a limitation: requiring motif analysis to distinguish direct binding events from indirect regulation through partner proteins (Bailey and MacHanick, 2012; Gordân et al., 2009). Additionally, ChIP-seq does not capture gene expression data, thus leaving out the context and intensity of the regulation that the TF confers. The valuable outputs of a ChIP-seq analysis have the potential to be paired with RNA-expression data to fully pinpoint TF function and the specific modulation of each gene (Angelini and Costa, 2014; Dresden et al., 2025; Muhammad et al., 2019). Furthermore, the technique requires high-quality, specific antibodies against the TF of interest or a transgenic line with a tagged TF, which can be challenging, time-consuming, and expensive to obtain, particularly for uncharacterized TFs or those in non-model plant species. The resolution of traditional ChIP-seq is inherently limited by chromatin fragmentation size and antibody specificity, typically resulting in binding peaks of several hundred base pairs rather than precise location of binding, but with new advances such as ChIP-nexus, the data gained from ChIP experiments has become more precise.

### 3.2 Restriction enzyme-mediated enhanced liberation sequencing

REEL-seq represents a new development in EMSA technology that offers a high-throughput discovery platform for TF characterization (Wu et al., 2022; Zhao et al., 2020). REEL-seq combines EMSA with next-generation sequencing to enable genome-wide identification of TF binding sites from gel-shift assays. This approach involves performing EMSA with genomic DNA libraries of DNA fragments, extracting protein-DNA complexes from the gel shift bands, and subjecting the recovered DNA to sequencing to potentially identify thousands of binding sites simultaneously. The method preserves EMSA’s fundamental advantage of detecting direct protein-DNA interactions under controlled *in vitro* conditions while expanding its discovery potential from single binding sites to genome-wide binding landscapes. The approach’s ability to work with purified recombinant proteins makes it ideal for studying plant TFs that may be difficult to express stably *in planta* or for which specific antibodies are not available.

However, REEL-seq shares several inherent limitations with the traditional EMSA approach. The *in vitro* nature of the assay means it cannot capture the influence of chromatin context, epigenetic modifications, or cofactor availability that regulate TF binding *in planta*, potentially identifying binding sites that are accessible *in vitro* but occluded or functionally irrelevant in the native cellular environment. Although it yields genome-wide discoveries, the method also cannot provide information about the functional consequences of binding events.

### 3.3 DNA adenine methyltransferase identification

DamID represents an alternative approach to ChIP for mapping protein-DNA interactions *in vivo*, utilizing adenine methylation-based tagging to identify genomic binding sites of TFs (Alvarez and Hinckley, 2023; Germann and Gaudin, 2011; Greil et al., 2006). The technique involves fusing the TF-of-interest to the prokaryotic Dam methyltransferase enzyme, which methylates adenine at the N6-position within GATC DNA sequences. When expressed in plant cells, the Dam fusion protein is tethered to the native genomic binding sites of the TF and deposits specific methylation “fingerprints” in the vicinity, which can then be detected and quantified using methylation-sensitive restriction enzymes.

The method offers several distinct advantages over traditional ChIP-seq approaches for TF characterization. DamID eliminates the need for high-quality, specific antibodies or DNA cross-linking procedures. Also, the covalent nature of methylation creates a stable fingerprint that can record transient, indirect, or weak protein-DNA associations that might be missed by ChIP-based methods. Additionally, DamID requires only standard molecular biology techniques including DNA extraction, enzymatic digestion, and quantitative PCR, making it less specialized than the chromatin preparation of ChIP-seq.

Some limitations of DamID for TF characterization include low-resolution mapping compared to modern ChIP-seq approaches, as methylation occurs within a several-hundred base pair radius around the binding site rather than pinpointing exact binding locations. DamID also requires very low expression levels of Dam fusion proteins to prevent saturation effects and maintain the ability to distinguish target from non-target regions, which can be technically challenging to achieve consistently. An additional plant-specific concern is the potential toxicity of Dam methyltransferase, which when highly expressed, can interfere with endogenous methylation patterns and normal plant development. Finally, like other binding-based methods, DamID provides no direct information about the functional consequences of protein-DNA interactions or the magnitude of gene expression changes resulting from TF binding. While DamID has been widely and successfully applied in animal systems, plant-specific implementations remain relatively limited, so careful evaluation of feasibility and potential toxicity in their species of interest is necessary before investing in this approach.

### 3.4 Cleavage under targets and tagmentation

CUT&Tag represents another advance in chromatin profiling technology which addresses limitations of traditional ChIP-seq while maintaining the advantage of studying TF binding within the native chromatin context. This approach combines antibody-directed targeting with Tn5 transposase-mediated tagmentation to map TF binding sites modifications from small cell populations (Kaya-Okur et al., 2019; Ouyang et al., 2021; Abbasova et al., 2025). In this method, a primary antibody recognizes the TF of interest, and a protein A–Tn5 fusion bound to a secondary antibody localizes the transposase to TF-bound chromatin. Upon activation, Tn5 simultaneously cleaves DNA adjacent to the TF and inserts sequencing adapters in a single tagmentation reaction, generating libraries enriched for fragments flanking TF binding sites with minimal handling. This streamlined workflow eliminates formaldehyde crosslinking, extensive chromatin fragmentation, and large input requirements while retaining high positional resolution of TF occupancy in native chromatin. The high-resolution nature of CUT&Tag enables location of TF binding sites relative to transcription start sites and other regulatory elements, providing detailed insights into the spatial organization and coordination of regulatory regions.

A compelling demonstration of CUT&Tag’s utility for plant TF characterization comes from a study that developed a slightly advanced version named biotinylated CUT&Tag (B-CUT&Tag) for profiling TFs in Arabidopsis (Tao et al., 2023). B-CUT&Tag was applied to map the genome-wide binding landscape of SPL9, a TF that plays crucial roles in plant development and flowering time regulation. The technique employs biotinylated Tn5 transposase complexed with biotin-modified adaptors, enabling subsequent magnetic bead purification that specifically isolates tagmented DNA fragments while removing the large amounts of intact, non-target chromatin that typically interfere with library construction and sequencing quality. This biotin purification step improves signal-to-noise ratios compared to standard CUT&Tag, making it particularly valuable for studying TFs with relatively low cellular abundance or fewer genome-wide binding sites.

CUT&Tag is closely related to CUT&RUN, which employs a protein A-MNase fusion for targeted chromatin digestion rather than Tn5 transposase-mediated tagmentation (Skene et al., 2018). Both methods offer high-resolution, low-input profiling capabilities, but CUT&Tag’s use of Tn5 transposase provides several advantages including streamlined library preparation, improved automation compatibility, and enhanced suitability for single-cell applications. Recent advances have extended CUT&Tag to single-cell profiling (scCUT&Tag), enabling investigation of TF binding heterogeneity within cell populations and providing insights into regulatory diversity at the cellular level (Bartosovic et al., 2021). These developments position CUT&Tag as a versatile platform for studying TF function across multiple scales of biological organization.

Despite the significant advantages, CUT&Tag faces several limitations of TF characterization that must be considered. Like ChIP-seq, the technique remains dependent on high-quality, specific antibodies or transgenic lines with tagged TFs, which can be challenging to obtain. CUT&Tag can be particularly complicated for profiling low-abundance TFs, where weak signals may be difficult to distinguish from background noise without highly sensitive reagents and optimized conditions. Additionally, the technique provides no direct information about gene expression consequences of TF binding, necessitating integration with RNA-seq or other expression profiling methods for comprehensive TF characterization.

### 3.5 DNA affinity purification sequencing and derivatives

DAP-seq represents an advancement in TF binding site identification that uses high-throughput sequencing like ChIP-seq but differs fundamentally in approach. The difference lies in using fragmented DNA instead of intact chromatin and bypassing the constraints of antibody availability or specificity.

DAP-seq operates through a streamlined workflow that begins with the construction of fusion proteins containing the DNA-binding domains of target TFs fused to affinity tags (Bartlett et al., 2017). These tagged TFs are expressed *in vitro* and subsequently used to capture genomic DNA fragments through affinity purification, followed by high-throughput sequencing to identify binding locations genome-wide. This approach has enabled large-scale mapping efforts, including the comprehensive characterization of 529 Arabidopsis TFs, demonstrating the technique’s remarkable scalability (O’Malley et al., 2016).

The fundamental advances of DAP-seq over ChIP-seq address several key limitations of the more traditional approach. DAP-seq eliminates the dependency on high-quality, specific antibodies, which can be challenging and expensive to obtain, particularly for newly discovered TFs or those in non-model plant species with the difficulty to produce transgenic lines with tagged TFs (Song, 2024). Affinity-tagged TFs are able to bind directly to naked genomic DNA fragments, creating higher resolution and higher likelihood of honing in on the motif that the TF binds within the DNA fragment. This precision is created by the removal of chromatin structure and other regulatory proteins, meaning that DAP-seq data reveals clear evidence of direct TF-DNA binding versus ChIP-seq data, which can include indirect and co-factor binding events. Furthermore, DAP-seq provides higher automation and reproducibility after successful fusion protein construction, reducing the experimental complexity and variability associated with ChIP-seq *in situ* protocols.

A compelling demonstration of DAP-seq’s transformative potential for TF characterization is exemplified by a comprehensive analysis of the maize AUXIN RESPONSE FACTOR (ARF) family (Galli et al., 2018). This study utilized DAP-seq to generate interaction maps for fourteen maize ARFs, successfully identifying 124,530 ARF binding sites across the maize genome. This example demonstrated the approach’s remarkable scalability by profiling multiple family members simultaneously, providing a comprehensive view of ARF binding specificity that would have been prohibitively expensive and time-consuming using traditional ChIP-seq approaches. The study provides compelling evidence for DAP-seq’s superior resolution in TF binding site identification, as the study successfully revealed distinct motif preferences between ARF clades that had not been previously characterized. Conversely, the research concluded that there were few differences in binding specificity, spacing, and target genes among ARF TFs within the same clade. The ability of DAP-seq to provide resolution at this level of detail allowed speculation about the importance of chromatin structure alteration in auxin-dependent responses rather than large differences in target binding sites. This level of motif discrimination exemplifies DAP-seq’s ability to pinpoint specific nucleotide sequences and confidently capture subtle but functionally important differences in DNA recognition between closely related TFs.

The DAP-seq platform has spawned several important derivatives that extend its capabilities to address specific research questions. Double DAP-seq (dDAP-seq) represents a significant advancement for studying synergistic DNA binding of interacting TFs, particularly focusing on the role of functional TF heterodimers. This approach has been successfully applied to profile twenty pairs of C/S1 bZIP heterodimers and S1 homodimers in Arabidopsis, providing insights into the molecular mechanisms underlying DNA binding and functional specificity of homo and heterodimers (Li et al., 2023) Another important derivative approach, multiDAP, enables comparative analyses across the genomes of multiple species in a single experiment. This provides insights on how TFs orchestrate similar functions across evolutionary lineages (Baumgart et al., 2021). In plants, optimized multiDAP protocols have been applied across diverse plant species to map TF binding sites genome-wide and quantify both deep conservation and rewiring of TF binding profiles among orthologous TFs. These datasets provide a powerful framework to study the evolutionary stability and divergence of TF binding, analogous to comparative TF binding studies that have been carried out in bacteria but now extended to complex plant genomes (Janky and van Helden, 2008).

DAP-seq also has limitations in TF characterization that must be addressed with other approaches. The most significant limitation is the absence of chromatin context. As mentioned previously, this aspect provides the positive of confirming direct binding, but this tradeoff results in missing important regulatory interactions that change based on chromatin accessibility, epigenetic modifications, or other protein co-factors. Consequently, DAP-seq may identify binding sites that are not functionally relevant *in planta*. Additionally, just as with ChIP-seq, DAP-seq lacks gene expression characterization. The functional consequences of TF binding and the specific modulation of gene expression is missed, necessitating integration with gene expression studies for comprehensive TF characterization (Yuan et al., 2024; Lai et al., 2020).

An important distinction among these binding methods is their capacity to detect transcription coregulators and cofactors, proteins that modulate TF function without directly binding DNA. Chromatin-based methods (ChIP-seq, CUT&Tag, CUT&RUN, and DamID) can capture these interactions when coregulators are part of functional TF complexes *in vivo*, such as the DELLA proteins that regulate gibberellin responses through protein–protein interactions with DNA-binding TFs (Fukazawa et al., 2014; Xue et al., 2022; Yoshida and Ueguchi-Tanaka, 2014). In contrast, *in vitro* approaches like traditional DAP-seq cannot detect cofactor-dependent interactions, although they excel at defining intrinsic DNA-binding specificity.

## 4 Gene expression analysis

Gene expression analysis represents a powerful functional genomics approach that can directly link TF activity to downstream changes in gene expression, filling knowledge gaps in TF characterization that binding-based methods alone cannot address. This functional perspective is essential for understanding how TFs translate their DNA binding capacity into actual gene regulatory effects. Whereas binding-based methods primarily quantify TF binding potential at regulatory DNA regions, gene expression approaches directly measure functional regulatory output, therein capturing the consequences of TF activity on the transcriptome. The characterization of TF function through genome-wide expression analysis can be achieved through multiple experimental approaches. Each offers distinct advantages for specific research questions, including loss of function or knock-out of TFs, stable over-expression lines, and rapid transient expression.

### 4.1 *In planta* gene expression analysis

Forward and reverse genetic approaches, including stable transformation and (un)targeted mutagenesis (EMS, CRISPR/Cas9), can generate transgenic or mutant lines that either knock-out or over-express the TF of interest, providing opportunities for detailed long-term, repeatable studies of TF function across developmental stages and environmental conditions. Both transgenic lines and chemically-induced mutants enable comprehensive phenotypic analysis and detailed characterization of TF effects on plant growth, development, and responses to the environment. These systems can be coupled with qRT-PCR and RNA-seq analysis to monitor gene expression changes (Cheng et al., 2022; Yu et al., 2022; Zhang et al., 2022). In some cases, alteration of the expression of a TF results in dramatic impacts on plant growth, disrupting normal plant development and function. Inducible over-expression systems address these potential drawbacks of constitutive TF expression by enabling temporal control over TF activity, thereby distinguishing between immediate and delayed, pleiotropic effects. The use of the estradiol-inducible constructs exemplifies the power of conditional TF over-expression for functional studies (Curtis and Grossniklaus, 2003; Zuo et al., 2000). This system provides precise temporal control over TF production and availability to bind. This helps eliminate developmental abnormalities that may result from constitutive TF overexpression, and enables studies in otherwise lethal or severely compromised transgenic lines (Husbands et al., 2023; Maki et al., 2019).

RNA-seq can be utilized in stable transformation studies to enable genome-wide analysis of TF regulatory effects, providing comprehensive insights into the scope and complexity of TF regulatory networks (Tu et al., 2022; Tyagi et al., 2022). The comprehensive nature of RNA-seq enables identification of unknown TF targets and reveals the full scope of regulatory networks controlled by specific TFs (Yun et al., 2024). This genome-wide perspective is essential for understanding how TFs coordinate complex biological processes and for identifying regulatory modules or co-regulated gene sets.

Genome-wide expression studies address several critical limitations inherent in binding-based TF characterization methods. While techniques like ChIP-seq and DAP-seq excel at identifying potential TF binding sites, they provide limited information about the functional relevance of these binding events or their impact on gene expression. This approach also provides insights into the hierarchical organization of GRNs by revealing both direct and indirect targets of TF regulation that have the potential to be separated by promoter binding motif analysis or further experimentation. While direct targets represent genes whose expression changes immediately upon TF binding, indirect targets reflect secondary effects mediated through the regulation of other TFs or regulatory factors. This view of TF function is essential for understanding how individual TFs coordinate effects that cascade through interconnected gene networks. Additionally, over-expression studies can reveal context-dependent aspects of TF function, as the same TF may regulate different sets of genes under different experimental conditions or developmental stages.

A recent demonstration of over-expression RNA-seq for TF characterization comes from the study of SLIM1 (Sulfur Limitation 1) in *Arabidopsis thaliana* (Apodiakou et al., 2024). SLIM1 is a plant-specific TF traditionally known for coordinating gene expression in response to sulfur deficiency, but its roles under nutrient-sufficient conditions remained poorly understood. The study employed constitutive SLIM1 over-expression combined with RNA-seq analysis to systematically characterize SLIM1’s regulatory network and functional roles beyond sulfur stress responses. The over-expression RNA-seq analysis revealed that SLIM1 regulates a remarkably broad network of 1,731 genes directly or indirectly, demonstrating the TF’s extensive regulatory influence. The study identified distinct temporal patterns of gene expression changes, with SLIM1 over-expression leading to earlier downregulation of photosynthesis-associated genes and earlier upregulation of senescence-associated genes compared to wild-type plants. This comprehensive transcriptome profiling revealed that SLIM1 functions as a key regulator of developmental timing, accelerating vegetative development and promoting earlier flowering. The functional characterization through overexpression RNA-seq provided insights that would have been impossible to obtain through binding-based methods alone. This example provides a comprehensive model for TF characterization through expression analysis, from genome-wide profiling to temporal pattern identification, connecting molecular mechanisms with physiological outcomes.

The limitations of gene expression studies must also be carefully considered when designing experiments and interpreting results. The most significant concern is the potential for over-expression artifacts, where artificially high TF levels may lead to non-physiological binding events or regulatory interactions that do not occur under normal conditions. These artifacts can result in the identification of false-positive targets or regulatory relationships that are not relevant to the TF’s natural function. The technique also captures extensive indirect effects, as over-expression-induced changes in gene expression can trigger secondary responses that obscure the typical direct regulatory targets of the TF. The technique also provides no information about the specific DNA sequences or regulatory elements through which the TF exerts its effects, necessitating integration with binding-based methods for complete mechanistic understanding of the difference between direct and indirect targets of the TF.

### 4.2 Transient assay reporting genome-wide effects of transcription factors

Protoplasts offer a unique simplified cellular environment that enables controlled gene expression studies. They can reduce some confounding variables present in transgenic overexpression lines, although they represent a mixture of cell types from the source tissue unless cell-type-specific markers or sorting are employed, and the isolation process itself may alter cellular physiology. This cellular isolation makes protoplasts particularly valuable for characterizing transcriptional effects of TFs swiftly, conducting transient gene expression assays within the timeframe of days and with minimal plant material. Transient expression systems like protoplast transformations provide rapid methods for initial TF characterization, enabling evaluation of regulatory effects within days rather than months required for stable transformation. These systems are particularly valuable for screening multiple TFs or testing different expression constructs before committing to more extensive stable transformation efforts.

TARGET represents an experimental framework that addresses limitations in TF characterization by integrating transient expression systems with comprehensive transcriptomic analysis and separating direct targets from indirect targets. This approach operates through the transient expression of TFs in plant protoplasts, followed by transcriptomic analysis to identify both direct and indirect target genes, providing detailed insights into TF regulatory networks (Bargmann et al., 2013). TARGET combines the controlled environment of protoplast systems with the comprehensive coverage of genome-wide expression profiling, enabling detailed characterization of TF function across regulatory hierarchies.

TARGET begins with the transient transfection of plant protoplasts with plasmid constructs containing the TF of interest fused to a glucocorticoid receptor (GR) domain under the control of a constitutive promoter. The GR-tagged TF remains sequestered in the cytoplasm until activated by dexamethasone (DEX) treatment which enables nuclear translocation and DNA binding activity, while cycloheximide (CHX) treatment is used to block protein synthesis and distinguish direct targets (genes that respond to DEX treatment even in the presence of CHX) from indirect targets (genes that require new protein synthesis and whose activation or repression is therefore blocked by CHX treatment). Total RNA is extracted and subjected to RNA-seq to quantify genome-wide transcript abundance, with the resulting expression data enabling distinction between immediate direct targets and delayed indirect targets through the controlled activation system. The protoplast-based system offers several advantages including uniform TF expression across all cells, elimination of tissue-specific confounding factors, and the ability to study TFs from any plant species in a standardized experimental context.

TARGET addresses limitations that constrain previously mentioned approaches. The technique bridges the gap between binding potential and functional regulation by yielding the ability to separate gene expression changes of direct versus indirect targets. This nuanced over-expression RNA-seq works to resolve the ambiguity of gene expression changes resulting from questions about the specific mechanisms of TF regulation.

A demonstration of the utility of TARGET for comprehensive TF characterization comes from the study of NLP7, a key TF in plant nitrogen signaling pathways (Alvarez et al., 2020). This investigation employed TARGET to systematically characterize NLP7’s regulatory network and its role in coordinating nitrogen-responsive gene expression in Arabidopsis. The TARGET analysis revealed that NLP7 regulates a comprehensive network of nitrogen-responsive genes, enabling clear distinction between direct and indirect targets. By performing a ChIP-seq on the nuclei of transfected protoplasts using anti-GR antibodies against the NLP7-GR fusion protein, this study also represented a comparison of TARGET’s ability to distinguish direct TF targets with the accuracy of previously completed *in planta* ChIP-chip binding assays.

The scalability and broad applicability of TARGET for comprehensive TF characterization have been demonstrated through large-scale studies of gene regulatory networks. Scaling up of the TARGET approach systematically characterized 33 nitrogen early-response TFs, collectively identifying direct regulated targets for 88% of nitrogen-responsive genes in Arabidopsis (Brooks et al., 2019). This study exemplified TARGET’s capacity for bulk analysis by processing up to 24 TF assays per day through innovations including pooled cell sorting and standardized controls. The comprehensive dataset revealed that each TF functions as both an inducer and repressor of distinct target gene sets, with *cis*-binding motifs often showing specificity for regulation directionality. The study also demonstrated how TARGET-validated experiments can be integrated with computational network inference to refine predicted GRNs.

Even with its integration of gene expression data with true binding events, TARGET has limitations that must be considered. There is the potential for transient expression artifacts, where artificially high TF levels or the protoplast environment may lead to non-physiological regulatory interactions. The isolation of protoplasts involves cell wall removal and may alter cellular physiology in ways that affect gene expression responses, potentially leading to artifacts that do not reflect TF function in intact tissues. The technique provides no information about the specific DNA sequences or regulatory elements through which TFs exert their effects, necessitating integration with binding-based methods for complete mechanistic understanding. Additionally, the transient expression system may not capture long-term or stable regulatory relationships that develop over extended time periods in intact plants. TARGET overall represents an advanced method of analyzing gene regulatory effects of TF overexpression, honing in on direct TF targets and guiding subsequent studies into binding mechanisms.

## 5 Computational TF characterization

The advancement of computational biology has revolutionized TF characterization by providing powerful tools that can predict binding sites, infer regulatory networks, and integrate diverse datasets without requiring extensive experimental validation. Numerous computational approaches have emerged for plant TF analysis. These tools collectively address different aspects of TF biology, from structural analysis to network inference to functional annotation. While each computational method offers unique advantages and limitations, here we describe three approaches that represent particularly powerful and complementary strategies for comprehensive TF characterization and have demonstrated success in plant systems: Plant Transcription Factor Binding Site Prediction (PTFSpot) for structure-based binding site prediction, GEne Network Inference with Ensemble of trees (GENIE3) for expression-based network inference, and ConnecTF for integrating validated interaction datasets. This section examines these three computational frameworks in detail, demonstrating how they can be implemented to provide comprehensive insights into TF function before experimental validation. Additional common tools for motif discovery and network inference are also discussed below.

### 5.1 Plant transcription factor binding site prediction

PTFSpot represents a computational approach developed specifically for plant TF analysis (Gupta et al., 2024). The system integrates 3D protein structure information of TFs with DNA sequence data, providing comprehensive understanding of TF-DNA interactions and achieving >90% accuracy without requiring species-specific training.

For characterizing a newly discovered TF, PTFSpot offers a systematic computational approach that provides comprehensive insights before experimental work begins. The system analyzes the structure of the novel TF to classify it into established TF families or identify it as potentially novel, examines DNA-binding domain architecture to predict recognized sequence patterns, and performs genome-wide scanning to identify potential binding sites with confidence scores. This analysis generates ranked lists of potential target genes while mapping specific regulatory regions including promoters and enhancers where the TF is most likely to bind.

The method’s greatest advantage lies in requiring only the TF protein sequence and genome assembly to begin characterization, enabling rapid hypothesis generation within hours rather than the months required for traditional experimental approaches. PTFSpot predictions can be directly integrated with other computational methods, serving as input for GENIE3 network inference or validation for gene expression experiments, making it particularly valuable for gene regulatory network research where experimental resources may be limited and newly discovered TFs require rapid initial characterization.

While PTFSpot excels at predicting where a TF can bind based on sequence and structure, it cannot predict context-specific binding influenced by chromatin accessibility or competitive binding, necessitating experimental validation of computational predictions through other approaches to confirm functional relevance in plant biology.

### 5.2 GEne network inference with ensemble of trees

GENIE3 (GEne Network Inference with Ensemble of trees) offers a machine learning framework for identifying regulatory targets of specific TFs using only genome-wide expression data, addressing the fundamental challenge of determining which genes are functionally regulated by a TF of interest without requiring specialized experimental protocols (Huynh-Thu et al., 2010). A time-series extension, dynamical GENIE3 (dynGENIE3), was later developed for temporal data analysis (Huynh-Thu and Geurts, 2018). The algorithm’s flexible input system is particularly well-suited for focused TF characterization studies, with the ability to accept predefined candidate regulators, making it ideal for analyzing individual TFs or TF families of interest. When characterizing a specific TF, GENIE3 uses regression tree ensembles to model the relationship between the TF’s expression pattern and all other genes in the dataset, generating importance scores that indicate the likelihood of regulatory relationships and enabling comprehensive evaluation of thousands of potential target genes simultaneously.

An example of GENIE3’s utility for specific TF characterization comes from the analysis of NAM-A1, a TF controlling senescence in wheat, where GENIE3 predicted 79 target genes from a comprehensive dataset of 850 publicly available RNA-seq samples covering diverse wheat tissues and developmental stages (Harrington et al., 2019). Validation against RNA-seq analysis of NAM-A1 mutant plants showed statistically significant enrichment of target genes compared to random TFs and demonstrated the algorithm’s ability to identify biologically relevant regulatory relationships.

Beyond GENIE3, a range of newer GRN inference methods has been developed for bulk and time-series gene expression data. Inference of large-scale gene regulatory networks based on multi-model fusion (LSGRN), for example, uses ensemble learning to scale GRN reconstruction to very high-dimensional datasets, while integrative random forest approaches such as Integrative random forest for gene regulatory network inference (iRafNet) explicitly combine prior network information with expression data, and transformer-based frameworks such as Gene regulatory network inference enhanced by transformer (TRENDY) incorporate temporal structure more directly (Petralia et al., 2015; Tian et al., 2025; Wu et al., 2023). Systematic benchmarking studies have evaluated these methods against reference networks, showing that relative performance depends strongly on dataset size, noise level, and regulatory complexity (Karamveer and Uzun, 2024; Nguyen et al., 2021; Pratapa et al., 2020). Together, these methods illustrate that GENIE3 should be viewed as a robust baseline within a broader and rapidly evolving GRN inference toolkit, rather than as a single default choice for all use cases.

GRN inference tools must be noted as highly sensitive to input quality and sampling; limited or condition-restricted expression datasets can yield unstable, context-specific predictions. Moreover, these methods typically have high false discovery rates, so inferred TF–target links should be treated as hypotheses that require experimental validation rather than definitive regulatory relationships. While these GRN inference models cannot definitively distinguish between direct and indirect regulatory relationships, potentially identifying genes regulated through intermediate factors rather than direct TF binding, they provide valuable capability for greatly narrowing the scope of a TF’s regulatory network and generating prioritized target lists for experimental validation. The algorithms’ scalability, flexibility, and proven success in plant research applications position them as valuable computational tools for understanding individual TF function in both model and non-model plant species.

### 5.3 ConnecTF

ConnecTF represents a comprehensive web-based platform that addresses a critical bottleneck in plant TF characterization by integrating diverse validated TF–target gene interaction datasets to build and refine GRNs (Brooks et al., 2021). The platform provides a species-independent framework that combines TF-binding data (ChIP-seq, DAP-seq), TF-regulation data (TARGET), TF-TF protein interactions, and cis-motif datasets through a user-friendly query interface. ConnecTF’s unique strength lies in its ability to perform automated analysis using experimentally validated TF–target interactions as standards to refine predicted networks, addressing the fundamental challenge of distinguishing between TF binding and functional regulation. The platform enables researchers to identify TF modes-of-action by integrating regulation and binding datasets, revealing whether TFs act directly through DNA binding or indirectly through protein-protein interactions with partner TFs. ConnecTF hosts validated TF–target interactions for Arabidopsis (3.7M interactions for 423 TFs), maize (839K interactions for 139 TFs), and rice (293K interactions for 26 TFs), while the open-source framework allows researchers to create customized instances for any species of interest. This comprehensive integration platform represents a significant advancement in connecting the growing wealth of TF–target validation datasets with practical network analysis tools for plant systems biology applications.

### 5.4 Broader computational landscape

Beyond the three platforms detailed above, the plant TF research community has access to a diverse ecosystem of computational tools and databases. For motif discovery and analysis, the Multiple Expectation-maximization for Motif Elicitation (MEME) Suite provides widely used *de novo* motif discovery and enrichment tools, Hypergeometric Optimization of Motif EnRichment (HOMER) offers motif finding optimized for ChIP-seq and other genomic data, and plant-focused platforms such as Plant Promoter Analysis (PlantPAN) and Regulatory Sequence Analysis Tools Plants (RSAT Plants) couple motif analysis with curated regulatory element annotations (Bailey et al., 2015; Chow et al., 2024; Hetzel et al., 2016; Santana-Garcia et al., 2022). Curated TF binding motif databases, including JASPAR, Plant Transcription Factor Database (PlantTFDB), and FootprintDB, supply experimentally supported or high-confidence TF binding profiles and predicted sites across many plant species (Castro-Mondragon et al., 2022; Jin et al., 2017; Sebastian and Contreras-Moreira, 2014). However, it must be noted that even when conserved cis-regulatory motifs can be identified, unambiguously assigning each motif to a specific TF or TF family remains challenging because many TFs share similar binding preferences and motif–TF relationships are often degenerate. Experimental validation therefore remains essential to confirm computational predictions and establish functional relevance.

## 6 Integrated approaches

The characterization of TFs has evolved from single-method approaches to sophisticated, multi-technique pipelines that leverage the complementary strengths of different experimental and computational strategies. No single method can resolve where a TF can bind and which genes it functionally regulates; integrated workflows are essential to connect binding potential, chromatin context, and regulatory output into coherent GRNs (**Table 1**). The future of TF research lies in developing integrated workflows that combine computational predictions, binding site identification, and functional analysis to provide comprehensive understanding of TF regulatory networks. In **Box 1**, we outline scenario-based workflows that illustrate how to combine approaches in practice.

Box 1 **Experimental frameworks for TF characterization across model, non-model, and cell-type-specific plant systems.**

Scenario 1: *de novo* TF in a model plant (e.g., Arabidopsis with stable transgenics)

**Discovery:** Use coexpression and network inference tools (e.g., PTFSpot, GENIE3) on publicly available or in-house expression datasets to nominate candidate targets and regulatory modules for the TF.
**Binding:** Generate an epitope-tagged TF line and perform ChIP-seq or CUT&Tag to obtain genome-wide binding profiles in relevant tissues or conditions.
**Function:** Profile transcriptomes by RNA-seq in loss-of-function mutants and/or inducible overexpression lines to distinguish regulatory effects.
**Validation:** For high-priority targets, use Y1H and EMSA to confirm direct TF binding to key promoters or enhancers.
**Network:** Integrate binding, expression, and predicted interactions with public GRN resources (e.g., ConnecTF) to position the TF within existing regulatory networks and identify upstream regulators and downstream modules.

Scenario 2: TF in a non-model crop without stable transgenics

**Discovery:** Apply GENIE3 or related GRN inference methods to public or in-house RNA-seq datasets to propose candidate targets and co-regulated gene sets.
**Binding:** Use DAP-seq with *in vitro*–expressed TF to map binding sites genome-wide without requiring a transgenic line or TF-specific antibodies.
**Function:** Employ transient expression systems such as TARGET or related protoplast-based assays to measure direct versus indirect TF regulation.
**Chromatin:** Perform ATAC-seq in the relevant tissue to identify accessible chromatin and intersect with DAP-seq peaks, prioritizing bound sites that are accessible and thus more likely to be functional *in vivo*.
**Validation:** Validate a focused subset of high-confidence targets using individual binding assays (e.g., EMSA, reporter assays) in heterologous or transient systems.
**Network:** Use multiDAP or related comparative approaches to compare binding motifs and targets with orthologous TFs in model species, helping transfer regulatory knowledge into the crop context.

Scenario 3: Cell-type-specific TF function

**Discovery:** Use spatial transcriptomics or high-resolution RNA-seq to define the TF’s expression domain and identify co-expressed gene modules in specific cell types.
**Binding:** Combine cell-type isolation with ChIP-seq or CUT&Tag to generate cell-type-specific TF binding maps.
**Function:** After TF perturbation (inducible overexpression, CRISPR, or knockout lines), perform scRNA-seq to resolve cell-type-specific transcriptional responses and distinguish regulatory effects within each cell population.
**Network:** Infer cell-type-specific GRNs from scRNA-seq data using tools such as SCENIC+ or related frameworks, integrating cell-type-specific binding information to constrain and interpret predicted regulatory edges.

These scenarios illustrate how different method combinations can be matched to biological questions and practical constraints, rather than relying on any single “best” assay. **Table 1** and **Figure 1** provide a side-by-side comparison of throughput, resolution, and resource requirements to further guide method selection across diverse plant systems.

A recent comprehensive characterization of the CCT39 TF in poplar exemplifies the power of integrated multi-technique approaches for elucidating TF regulatory mechanisms (Chen et al., 2025). The study began with stable overexpression of *PpnCCT39* in transgenic poplar lines, which revealed striking phenotypic alterations including increased chlorophyll content, enhanced photosynthesis rates, altered leaf morphology, and modified chloroplast structure. These observations established the biological significance of the TF and guided subsequent molecular investigations. To identify when and where *PpnCCT39* exerts its regulatory effects, RNA-seq was performed across terminal buds and leaves at multiple developmental stages, revealing that *PpnCCT39* predominantly functions in young developing leaves rather than mature tissues. ChIP-seq analysis on the overexpression lines then mapped 17,194 potential direct regulatory target genes genome-wide, providing a comprehensive binding landscape that identified candidate genes involved in chlorophyll biosynthesis and photosynthesis pathways. The integration of RNA-seq and ChIP-seq datasets compared which genes were both differentially expressed in overexpression lines and directly bound by *PpnCCT39*, narrowing the large pool of candidates to high-confidence direct targets. Y1H validation assays were then employed to experimentally confirm that *PpnCCT39* directly binds to and activates three key genes which encode enzymes in the chlorophyll biosynthesis and photosynthesis pathways. This integrated workflow that progressed from phenotypic observation to expression profiling to genome-wide binding identification to targeted validation demonstrates how complementary techniques converge to provide mechanistic insights that no single method could achieve, ultimately revealing that *PpnCCT39* promotes chlorophyll biosynthesis and photosynthesis through direct transcriptional activation of specific metabolic genes.

## 7 Emerging directions

The experimental techniques described in Section 2–5 focus on bulk tissue assays that average signals across diverse cell populations and often infer TF activity indirectly. The field is rapidly moving toward higher-resolution approaches, including single-cell and spatial technologies that address cell-type heterogeneity and enable precise mapping of cell-type-specific regulatory networks. In this context, pooled high-throughput platforms such as Enrichment of Nuclear Trans-elements Reporter Assay in Plants (ENTRAP-seq) and Protoplast Isolation after Virus Overexpression in planTa (PIVOT) provide “single-cell-like” regulatory readouts from bulk nuclei or protoplast populations, while emerging chromatin accessibility and single-cell multi-omic assays deliver true cellular and spatial resolution. In parallel, systematic characterization of TF effector domains is beginning to bridge binding-site maps and functional transcriptional output. Here we briefly highlight these emerging technologies, which together represent the current frontier of plant TF research.

### 7.1 Enrichment of nuclear trans-elements reporter assay in plants

ENTRAP-seq is an example of this shift that enables testing of thousands of TF variants at once directly in plant cells (Alamos et al., 2025). This technique represents a pooled, high-throughput assay that introduces protein-coding libraries into plant cells and measures reporter activity in isolated nuclei, rather than intact single cells. This design enables massively parallel screening of thousands of TF variants in a bulk population while retaining limited cellular context through nucleus-level resolution of regulatory activity. By introducing libraries of these variants into plant leaves using agroinfiltration and pairing them with a fluorescent nuclear reporter, ENTRAP-seq allows for sorting nuclei based on reporter activation via magnetic separation. Because the gene delivery is done at low density, each cell typically receives just one TF variant, minimizing unwanted overlap of TF overexpression. High-throughput sequencing then measures how much each variant enriches in the “activated” nuclei, providing a direct, quantitative measure of its effect on gene expression. The functional output is also benchmarked with traditional fluorescence assays, ensuring robust results. This approach reveals subtle differences in the regulatory power of TFs, supports systematic mutation scans, and enables large-scale surveys of both synthetic and natural TFs. In short, ENTRAP-seq links what a TF does in its true plant cellular environment to its specific sequence identities, a longstanding challenge for the field. However, ENTRAP-seq remains limited by its reliance on pooled nuclei rather than intact tissues, the artificial reporter context, and the need to overexpress TF variants, which may not fully capture endogenous, cell-type-specific TF function.

### 7.2 Protoplast isolation after virus overexpression in planTa

PIVOT brings pooled, high-throughput screening to the single-cell level within intact plant tissue (Lowensohn et al., 2025). Like ENTRAP-seq, PIVOT operates on bulk protoplast populations rather than isolated single cells, but achieves single-cell level readouts by assigning viral barcodes to individual nuclei. This positions PIVOT as a transitional platform between traditional bulk assays and emerging true single-cell sequencing approaches. The method works by first infiltrating plant leaves with a pooled viral library where each viral vector carries a different TF along with a unique DNA barcode for identification. It overcomes a major limitation of pooled library screens by exploiting viral superinfection exclusion, a natural phenomenon where cells infected with an initial virus become resistant to subsequent viral infections, ensuring that each individual cell expresses only one TF from the pooled library.

Like Y1H systems that test TF-DNA interactions in yeast cells, PIVOT tests whether specific TFs can activate target promoters, but does so within the native plant cellular environment if the approach can be optimized for the chosen plant species. The experimental setup places a reporter gene (encoding a detectable surface marker) under the control of a promoter sequence of interest, similar to the use of reporter genes like HIS3 or LacZ under control of DNA bait sequences in Y1H. After viral delivery and TF expression, protoplasts are prepared from the infiltrated tissue. These protoplasts are then magnetically sorted based on whether they display the surface marker, indicating successful pathway activation by the expressed TF.

High-throughput sequencing of the DNA barcodes in the marker-positive versus marker-negative populations reveals which TFs from the library can activate the target promoter. Unlike Y1H, which requires validation in plant systems after yeast-based discovery, PIVOT directly identifies functional TF-promoter interactions within plant cells. This approach allows characterization of both expected and novel pathway regulators while avoiding confounding effects from gene redundancy or cellular stress that might occur in whole-plant over-expression studies, making it a scalable method for testing large TF libraries at true single-cell resolution within plant tissue. PIVOT’s limitations include its dependency on efficient host-dependent viral delivery in protoplast populations, usage of a synthetic reporter system, and inference of “single-cell-like” responses from barcode-resolved nuclei, so it cannot fully recapitulate native tissue architecture or the complete spectrum of in planta TF activities.

### 7.3 Chromatin accessibility and single-cell multi-omics

True single-cell and spatially resolved technologies represent the next frontier for understanding TF function in plant systems, moving beyond bulk assays that average signals across heterogeneous tissues. Chromatin accessibility profiling through Assay for Transposase-Accessible Chromatin with sequencing (ATAC-seq) and closely related DNase I–hypersensitive site assays identify accessible regulatory regions genome-wide and enables TF binding inference through footprint analysis, in which TF-protected sequences are detected within accessible chromatin (Buenrostro et al., 2015; Song and Crawford, 2010). In bulk tissue, these methods reveal the landscape of active regulatory elements, whereas single-cell ATAC-seq (scATAC-seq) enables cell-type-specific chromatin accessibility profiling (Chen et al., 2018).

ATAC-seq measures chromatin accessibility by using a hyperactive Tn5 transposase to insert sequencing adapters into regions of open chromatin. After transposition, the tagged DNA fragments are amplified and sequenced, and regions with high read density correspond to accessible regulatory elements such as promoters and enhancers. scATAC-seq extends this principle by profiling Tn5-tagged fragments from thousands of individual nuclei in parallel, enabling reconstruction of cell-type-specific accessibility profiles and TF motif enrichment across heterogeneous tissues.

A study in maize inflorescence meristems used scATAC-seq to examine how the dominant *Barren inflorescence3* (*Bif3*) mutant, which overexpresses the homeodomain TF ZmWUS1, alters chromatin accessibility in the stem-cell-rich core region of the inflorescence meristem (Bang et al., 2025). Regions of the chromatin with increased accessibility in *Bif3* were enriched for a known WUS-binding homeodomain recognition site. This motif enrichment supports a model in which overexpressed ZmWUS1 functions as a transcriptional activator that opens chromatin at target loci, directly linking TF activity to changes in chromatin state.

Integration of scRNA-seq with scATAC-seq in the same cells (single-cell multi-omics) enables unprecedented resolution in understanding TF regulatory mechanisms (Tang et al., 2009). A recent study generated a spatially resolved multi-omic single-cell atlas of soybean development, integrating gene expression and chromatin accessibility across ten tissues and over one hundred cell types (Zhang et al., 2025). This approach enabled prediction of TF regulatory modes (activator versus repressor function) in specific cell types by correlating TF expression, target gene expression, and accessibility at target loci, revealing not just where TFs bind, but how they function in precise developmental and spatial contexts.

These chromatin-based and single-cell approaches complement the bulk binding and expression methods detailed in earlier sections by providing critical spatial, temporal, and cell-type context that bulk assays cannot capture. As these technologies become more accessible and cost-effective for plant systems, they will enable construction of cell-type-specific regulatory networks and reveal how TFs coordinate development and environmental responses at unprecedented resolution.

Looking further ahead, adaption of Perturb-seq–style approaches hold strong promise for plant TF research (Dixit et al., 2016). In Perturb-seq, pooled CRISPR-based perturbations (for example, TF knockdown, knockout, or specific domain mutations) are introduced into a population of cells, and each perturbation is tagged with a unique guide barcode that is captured together with the transcriptome by scRNA-seq. This design enables simultaneous measurement of thousands of single-cell transcriptomes, each linked to a defined genetic perturbation, allowing systematic, high-throughput dissection of TF function and regulatory networks at single-cell resolution directly in planta. Such strategies could enable systematic functional screening of TF variants or candidate targets at single-cell resolution directly in planta, providing a powerful complement to the observational single-cell and multi-omic assays described above. Additionally, computational methods specifically designed to infer gene regulatory networks from scRNA-seq data, such as SCENIC+, complement bulk-focused network inference tools and offer new opportunities to map cell-type-specific regulatory logic as plant single-cell datasets accumulate (Bravo González-Blas et al., 2023).

Despite their transformative potential, chromatin accessibility and single-cell multi-omic assays remain limited by high cost, substantial technical and computational demands, and challenges in integrating sparse, noisy signals into biologically interpretable TF–target relationships. Important to note, chromatin accessibility and TF motif enrichment report regulatory potential; linking these profiles to perturbed expression or reporter assays is critical to establish functional TF–target relationships. Also, even with single-cell and multi-omic data, reconstructed networks from these techniques remain snapshots of particular conditions, emphasizing the need to sample diverse tissues, developmental stages, and environments.

### 7.4 TF effector domain characterization

An emerging area bridging TF binding and functional regulation is the systematic characterization of transcriptional effector domains, the protein regions that recruit transcriptional machinery or chromatin regulators to activate or repress target genes. Recent high-throughput approaches have enabled comprehensive mapping of these domains in plant TFs. Moffrey et al. developed a systematic approach to identify minimal transcriptional activation and repression domains across plant TF families, revealing that short peptide sequences on the order of 10–30 amino acids can be sufficient for transcriptional output Morffy et al. (2024). Hummel et al. used a massively parallel reporter assay to quantify the regulatory activity of thousands of TF domain variants simultaneously in plant cells, defining sequence features associated with activation and repression strength (Hummel et al. (2024).

These effector domain characterization approaches complement binding-site identification methods by directly measuring transcriptional output. They help determine whether TFs act primarily as activators, repressors, or context-dependent dual regulators, and they enable engineering of synthetic TFs with defined regulatory properties. Integrating effector domain mapping with binding site identification (e.g., DAP-seq, ChIP-seq) and target gene expression profiling (e.g., RNA-seq, TARGET) provides a more complete view of TF mechanism from DNA recognition through to transcriptional output.

By integrating these next-generation single-cell and spatial approaches with systematic effector domain mapping, plant TF research is poised to move beyond bulk assays toward quantitative, scalable mapping of GRNs directly within their native cellular context. Together, these methods promise both more precise synthetic biology tools and a deeper mechanistic understanding of how transcriptional networks are wired and executed in plants.

## 8 Conclusion

New TF characterization techniques continuously emerge, each with distinct capabilities, requirements, and limitations. In particular, the field of computational approaches is rapidly developing, providing the benefit of more efficient, in-depth data analysis and supplementing wet-lab techniques. By understanding the evolution of these methods and their respective strengths and limitations, experimental strategies can be designed that maximize biological insights while efficiently utilizing available resources. It should be emphasized that no single method provides complete answers about TF function. Instead, the most powerful insights emerge from integrating multiple approaches: combining genome-wide binding data with functional studies and leveraging computational predictions with experimental validation. As the field continues to evolve, this integrative philosophy will become increasingly important for unlocking the full potential of TFs for plant improvement. This review provides a comprehensive roadmap for researchers navigating the expanding toolkit of TF characterization methods, empowering informed decisions that transform individual TF discoveries into mechanistic understanding of gene regulatory networks and, ultimately, into targeted strategies for crop enhancement and agricultural innovation.
